# Phylogeny and Polyploidy Evolution of the Suckers (Teleostei: Catostomidae)

**DOI:** 10.3390/biology13121072

**Published:** 2024-12-20

**Authors:** Lei Yang, Richard L. Mayden, Gavin J. P. Naylor

**Affiliations:** 1 Florida Museum of Natural History, University of Florida, 1659 Museum Rd., Gainesville, FL 32611, USA; 2 Biology Department, Saint Louis University, 3507 Laclede Avenue, St. Louis, MO 63103, USA

**Keywords:** gene duplication, genome, mitogenome, NGS, nuclear, paralog

## Abstract

Fishes in the cypriniform family Catostomidae (suckers) are evolutionary tetraploids. The use of nuclear markers in the phylogenetic study of this important group has been greatly hindered by the challenge of identifying paralogous copies of genes. In the present study, we used two different methods to separate the gene copies of five single-copy nuclear genes (i.e., RAG1, EGR2B, EGR3, IRBP2, and RAG2). For each gene, all sequences of Copy I formed a clade that was sister to the clade formed by all sequences of Copy II in the phylogenetic trees. The maternal and paternal progenitor of the tetraploid ancestor of the Catostomidae could not be determined. We also constructed a mitochondrial tree to reflect the maternal relationships among major catostomid lineages. Our data appear to support a sister relationship between Catostominae and a monophyletic group composed of Myxocyprininae, Cycleptinae, and Ictiobinae. However, within Catostominae, there is significant conflict between mitochondrial and nuclear data regarding the relationships among Erimyzonini, Catostomini, and Moxostomatini/Thoburnini. Many indels, unexpected stop codons, and possible gene loss were identified in one gene copy of RAG1, RAG2, and IRBP2. We believe that additional nuclear genome data are needed to better resolve the phylogenetic relationships within the family Catostomidae.

## Simple Summary

Polyploids possess more than two complete sets of chromosomes. While common in plants, they are found in only a few clades of fishes. Catostomidae is a freshwater fish family composed exclusively of tetraploids. Studying the relationships among members of this family has been challenging. In this study, we collected DNA sequence data for five nuclear genes, separated the gene copies, and provided new insights into the evolution of this polyploid lineage.

## 1. Introduction

Many organisms possess three or more sets of chromosomes, a phenomenon known as polyploidy or whole-genome duplication [1,2]. Polyploidy is widely recognized as an important evolutionary and ecological force across plants, animals, and other organisms [3,4,5]. Numerous studies have explored various aspects of polyploidy, e.g., phylogenetics [6], evolution [7], biogeography [8], and ecology [9].

The family Catostomidae (suckers) is one of the few vertebrate lineages that are comprised exclusively of polyploids [1,10]. It belongs to the order Cypriniformes and contains 85 described freshwater species in 15 genera [11]. All but two species (*Myxocyprinus asiaticus* and *Catostomus catostomus*) are native to North America [12]. Uyeno and Smith (1972) proposed that catostomid fishes have a tetraploid origin which was caused by a hybridization event early in the evolutionary history of this group [13]. There are four sets of nuclear genomes in catostomid fishes and, theoretically, each single-copy nuclear gene should have two gene copies. Surprisingly, few studies have tried to separate nuclear gene copies from species of these fishes. Relatively more such studies have been performed for polyploids of other cypriniform groups, e.g., common carp, goldfish and their cyprinine allies [14], and loaches [15]. Bart et al. (2010) did separate two copies of the growth hormone gene from some species of catostomid fishes [16]. However, it seems that the nuclear gene they examined is not single-copy but rather from a gene family. In the present study, we chose to use five nuclear genes that are widely used in phylogenetic studies on cypriniform fishes and are considered single-copy genes. The five nuclear genes are RAG1 (recombination activating gene 1, exon 3), EGR2B (early growth response protein 2B gene), EGR3 (early growth response protein 3 gene), IRBP2 (interphotoreceptor retinoid binding protein gene 2), and RAG2 (recombination activating gene 2). A single-copy nuclear gene should have one copy (2 alleles) in diploids, two copies (4 alleles) in tetraploids, and three copies (6 alleles) in hexaploids. Single-copy nuclear genes can be used to trace the evolutionary histories of ohnologs resulting from whole genome duplication events, while minimizing the influence of paralogs resulting from any other smaller-scale gene duplication events [17].

Even though several studies have been conducted to investigate the phylogenetic relationships within the family Catostomidae (e.g., [16,18,19,20,21,22,23]), some disagreements still exist regarding the inter-relationships among the constituent tribes/subfamilies. One of the main disagreements is whether Ictiobinae, Cycleptinae, and Myxocyprininae form a monophyletic group, and if they do not, the relationships among these taxa and the Catostominae. Moreover, within the subfamily Catostominae, although the sister relationships between the tribes Moxostomatini and Thoburniini were supported in most analyses, the relationships among Catostomini, Erimyzonini, and Moxostomatini/Thoburniini vary from study to study. Early molecular studies relied heavily on mitochondrial genes (e.g., [21]). More recent studies began to use nuclear genes [22] or both mitochondrial and nuclear genes [23]. The use of nuclear markers in phylogenetic studies of the family Catostomidae has been hampered by the difficulties associated with the identification of orthologous copies of corresponding genes. If nuclear gene copies are not sorted appropriately, homology cannot be confidently established, potentially misleading phylogenetic inferences. This may have compromised the accuracy of the phylogenetic studies of the Catostomidae in the past. Chen and Mayden (2012) used a single nuclear gene (IRBP2), and the authors claimed that the primers they used are copy-specific and the sequences they generated for the catostomids are orthologs [22]. Bagley et al. (2018) did not validate the orthology of gene copies for the three nuclear genes they used [23]. Krabbenhoft et al. (2021) [24] and Liu et al. (2022) [25] published the whole genome sequencing results of two different individuals of Chinese sucker (*Myxocyprinus asiaticus*). However, more genera and species need to be sequenced before a phylogeny of Catostomidae can be inferred based on whole genome sequences.

Rothfels et al. (2017) summarized different approaches that have been used to separate multiple nuclear gene copies when studying polyploidy or gene family evolution [26]. Based on the information from that study, these approaches can roughly be grouped into the following four categories: (1) PCR (Polymerase Chain Reaction) amplicon cloning (e.g., [27]); (2) using copy-specific primers (e.g., [28]); (3) single-molecule PCR (e.g., [29]); (4) Next-Generation Sequencing (NGS) or third-generation sequencing followed by data phasing (e.g., [26]). In the past, the first approach has been most frequently adopted by researchers. Approaches in the last category were not available until a little over a decade ago. They are based on several different sequencing platforms (e.g., Roche 454, Illumina, and PacBio) and various pipelines have been developed to detect and assemble multiple nuclear gene copies from large amount of short or long sequencing reads (e.g., [26,30,31]). In the current study, we used two different methods to separate gene copies in the catostomid fishes: DNA cloning followed by data phasing and Next-Generation Sequencing (NGS) followed by data phasing.

The objectives of this study are two-fold: (1) to investigate the phylogenetic relationships among major lineages of Catostomidae using both mitochondrial and nuclear genes; (2) to study the polyploidy evolution of this family through separating the two homoeologous copies of single-copy nuclear genes.

## 2. Materials and Methods

### 2.1. Ethics Statement

Archived tissue samples from museums were used for this project. No live animals were intentionally sampled. Therefore, no ethical approval was required.

### 2.2. Taxon Sampling and DNA Extraction

In this study, new mitochondrial and nuclear sequence data were collected from 35 and 26 catostomid species, respectively. Most samples were from the tissue collection of Saint Louis University and the Florida Museum of Natural History at University of Florida. Some tissue samples were kindly provided by Tulane University, Kansas University and Oregon State University. The detailed sample information can be found in Tables S1 and S2. Genomic DNA was extracted from fin clips or muscle tissue (stored at 95–99% ethanol) with the DNeasy Blood & Tissue Kit (Qiagen Sciences Inc., Germantown, MD, USA) or the E.Z.N.A. Tissue DNA Kit (Omega Bio-Tek, Inc., Norcross, GA, USA), according to manufacturers’ protocols.

### 2.3. The Mitochondrial Dataset

A total of 35 whole mitogenome sequences were newly determined from catostomid samples using a gene capture method followed by Next-Generation Sequencing on an Illumina MiSeq platform [32]. We also downloaded the whole or partial mitogenome sequence data for 16 other species of Catostomidae, forty-three species of other cypriniform fishes, and three species of the orders Gonorynchiformes, Siluriformes, and Characiformes from GenBank (Table S1). Mitochondrial genomic sequences were aligned following Saitoh et al. (2006) [33]. The complementary strand sequences were used for L-strand-encoded genes (ND6 and eight tRNA genes). The final alignment contained 97 species and comprising 14,888 nucleotide sites, including 11,337 sites from 13 protein-coding genes, 2096 sites (stems: 1173 sites; loops: 923 sites) from two rRNA genes, and 1401 sites (stems: 914 sites; loops: 487 sites) from 22 tRNA genes (Table 1).

### 2.4. The Nuclear RAG1 Dataset

PCR amplifications of the RAG1 gene were carried out in 25 μL reactions (2.5 μL [5×] reaction buffer, 2 μL dNTP [2.5 mM each], 2 μL MgCl_2_ (25 mM), 0.5 μL [10 μM] each primer, 4 μL template DNA [~10 ng/μL], and 0.1 μL ExTaq Taq DNA polymerase (Takara, Osaka, Japan)). The following thermal cycling profiles were adopted: 95 °C pre-denaturing (4 min), 95 °C denaturing (40 s), 53 °C annealing (40 s), 72 °C extension (90 s), for 30 cycles, and 72 °C final extension (7 min). Multiple sets of PCR primers were used to try to amplify all copies of the RAG1 fragment existed in a sample. All primer sets share the same forward primer, which is R1 2533F (5′-CTG AGC TGC AGT CAG TAC CAT AAG ATG T-3′; [34]). The reverse primers used include R1 4090R (5′-CTG AGT CCT TGT GAG CTT CCA TRA AYT T-3′; [34]), R1 4078R (5′-TGA GCC TCC ATG AAC TTC TGA AGR TAY TT-3′; [34]), and R1 4061R (5′-AAT ACT TGG AGG TGT AGA GCC AGT-3′; [35]). After PCR amplifications, two methods have been used to get the DNA sequences of the two RAG1 copies: DNA cloning followed by data phasing (Method 1) and Next-Generation Sequencing followed by data phasing (Method 2).

Method 1: We cloned and sequenced the nuclear RAG1 gene for 14 catostomid species. The PCR products were ligated into pGEM-T vector (Promega, Madison, WI, USA) and cloned into JM109 competent *Escherichia coli* (Promega, Madison, WI, USA). The standard blue–white colony screening was performed following cloning and incubation. We picked up to 26 positive clones for each sample and these clones were then used as templates for second-round PCR amplifications, also using 30 cycles. The PCR products were then purified with ExoSAP-IT (Applied Biosystems, Waltham, MA, USA). Both the purification and sequencing were conducted at the htSEQ High-Throughput Genomics Unit (University of Washington, Seattle, WA, USA). Primers used for PCR amplifications were also used for sequencing. SeaView v. 5.0.4 [36] is used to clean up and then align the Sanger sequences of all sequenced clones of a sample. The homoeologous copies of RAG1 were sorted out and determined after removing chimeric sequences from the alignment. During the above phasing process, we have temporarily reduced the alignment to include only the variable sites to make the process easier.

Method 2: For some species, we tried a different method to obtain the DNA sequences of the two homoeologous copies of the RAG1 fragment. The PCR amplicon of RAG1 of each sample was sheared to c.500 bp using acoustic ultrasonication on a Covaris M220 Focused-ultrasonicator (Covaris, Inc., Woburn, MA, USA). An Illumina sequencing library [37] was then prepared for each sample using the “with-bead” method [38], following [39]. The libraries were indexed through PCR amplification (number of cycles = 5). The indexed libraries were pooled in equimolar ratios and pooled libraries were diluted to 12–15 pM for paired-end 300 bp sequencing on an Illumina MiSeq benchtop sequencer (Illumina, Inc., San Diego, CA, USA) at the Interdisciplinary Center for Biotechnology Research (ICBR) of University of Florida.

The sequence reads of each species were identified and sorted by their respective indices once the sequencing was finished. Adapters and low-quality reads were removed using cutadapt and FastQC embedded in Trim Galore! v0.4.3 [40]. To reduce file sizes and make the downstream analyses easier, in some cases, the reads to be retained were limited to those that have a read length equal to or larger than a set value (e.g., 250). The retained reads were imported into Geneious Pro v11.0.5 (Biomatters Ltd., Auckland, New Zealand. Available at http://www.geneious.com). Duplicated reads were then removed. The resulting reads of each species were mapped to the Sanger-sequenced RAG1 sequence of *Erimyzon oblongus* downloaded from GenBank (accession number: EU711117). After that, we deleted the reference sequence and the nucleotides that mapped beyond the boundaries defined by the sequences of the forward and reverse primers used for RAG1 amplification. We then exported the large read alignment as a FASTA format file. The R package *copyseparator* 1.2.0 [41] was then used to separate and assemble the two gene copies (when present) from the imported FASTA alignment (key steps illustrated in Figure S1). The function “sep_assem” was used and the parameter “copy_number” was set as 2, “read_length” as 300, and “overlap” as 225. More information on how *copyseparator* works and its limitations can be found on the GitHub page of this package through CRAN (The Comprehensive R Archive Network). For each assembled gene copy, we also manually checked the assembling process to ensure that no chimeric sequences were created.

We noticed that, in most cases, it is impossible to separate the two alleles of a gene copy. That is because the allele sequences are usually very similar to each other and the short Illumina read sequences do not exhibit enough variability to be linked together through their polymorphic sites. In this study, we chose to separate gene copies (homoeologs) only. As a result, each gene copy sequence may contain a few ambiguous sites (e.g., R, Y, M, or K) to reflect the existence of allelic variation.

The final RAG1 dataset contains representatives of all 15 currently recognized genera and 26 valid species (Table 1). To better represent each genus, in most cases, the type species are included. Eighteen species were used as outgroups and their RAG1 sequences were downloaded from GenBank. See Table S2 for accession numbers for all sequences included in this study. The final alignment of the RAG1 dataset is 1497 bp in length (Table 1).

### 2.5. The Nuclear EGR2B, EGR3, IRBP2, and RAG2 Datasets

For 19 catostomid species (see Table S2), we also obtained the DNA sequences for the nuclear genes EGR2B, EGR3, IRBP2, and RAG2. Primers and protocols used for PCR amplification and Sanger sequencing of the first three genes can be found in [42]. For RAG2, the primer set RAG2-f1 and RAG2-r6 was used for both PCR amplification and Sanger sequencing. The primer sequences and detailed PCR protocol can be found in [43]. The above-mentioned Method 2 was used to obtain the Illumina reads from PCR amplicons and to separate the two homoeologous copies of each nuclear gene for each sample. We usually pooled the PCR amplicons of several nuclear genes together based on the brightness of bands on the agarose gel and prepared a single dual-indexed NGS library for each sample. For read mapping in Geneious, the reference is a single FASTA file that contains the following sequences downloaded from GenBank: EU409734 (EGR2B; *Catostomus commersonii*), EU409766 (EGR3; *C. commersonii*), JX488958 (IRBP2; *C. commersonii*), and DQ367043 (RAG2; *Myxocyprinus asiaticus*). The reference sequence for RAG1 (EU711117; *Erimyzon oblongus*) was also added when RAG1 amplicons were represented in the reads. In Geneious, the mapping results for each gene were separated automatically. After deleting the reference sequences and the nucleotides not directly mapped to the references, the resulting FASTA file for each gene was saved to a folder to be processed one by one using the R package *copyseparator* 1.2.0.

The final EGR2B, EGR3, IRBP2, and RAG2 datasets were 846 bp, 953 bp, 864 bp, and 1314 bp in length, respectively (Table 1). They all contain 19 catostomid species but contain different numbers (10–26) of outgroups whose sequences were downloaded from GenBank (Table 1 and Table S2).

### 2.6. The Concatenated Gene Dataset

We also built a “7-nuclear dataset” by putting together the Copy I sequences of the five nuclear gene and the Copy II sequences of EGR2B and EGR3. The Copy II sequences of the RAG1, RAG2, and IRBP2 genes were excluded from this dataset because they were either not identified or contained a significant amount of indels and/or unexpected stop codons in some species. The Copy I and Copy II of both EGR2B and EGR3 were treated as four different genes and be concatenated because, based on our preliminary results, the whole genome duplication event in Catostomidae likely preceded the common ancestor of all members of this family. We also built an “All-gene dataset” by adding corresponding mitochondrial sequences to the 7-nuclear dataset. Both the “7-nuclear dataset” and the “All-gene dataset” contain 34 species (19 catostomid species and 15 outgroups). The former is 7237 bp in length, while the latter is 22,125 bp in length (Table 1).

### 2.7. Phylogenetic Analyses

MITOCHONDRIAL DATASET Partitioned Maximum Likelihood (ML) and bootstrap analyses (MLBP) were conducted for the Mitochondrial dataset using RAxML v.8.2.12 [44,45]. PartitionFinder v2.1.1 [46] was used to determine the best partitioning scheme and model of nucleotide substitution. A total of 200 distinct runs were performed based on 200 random starting trees using the default settings of the program. The tree with the best likelihood score was chosen as the final tree. The non-parametric bootstrap analyses (1000 replicates) were also conducted using RAxML [47] with the same partitioning strategy and nucleotide substitution model as above. PAUP 4.0.b10 [48] was then used to obtain the 50% majority rule consensus tree and bootstrap values (BP).

NUCLEAR DATASETS Partitioned ML and MLBP analyses were conducted for each of the five individual nuclear gene datasets using RAxML. The individual datasets were partitioned by codon positions. For the ML analyses, a total of 1000 rather than 200 distinct runs were performed for each dataset.

CONCATENATED DATASETS Partitioned ML and MLBP analyses were conducted for the “7-nuclear dataset” and the “All-gene dataset” following the same procedure described above for the Mitochondrial dataset. For the ML analyses, a total of 200 distinct runs were performed for each dataset. In this study, we chose to build trees from concatenated datasets rather than using a coalescent-based species tree inference method. This is because both the Summary statistics species tree methods (e.g., MP-EST [49], ASTRAL [50]) and the Full data species tree methods (e.g., SVDQuartets [51]) usually need data from numerous loci to perform well.

### 2.8. Nuclear Genes’ Locations on Genomes

Currently, there are nuclear reference genomes for four species of catostomids available in the GenBank, i.e., *Myxocyprinus asiaticus* (GenBank: GCA_019703515.2), *Xyrauchen texanus* (GenBank: GCA_025860055.1), *Moxostoma hubbsi* (GenBank: GCA_032164185.1), and *Catostomus latipinnis* (GenBank: GCA_036785435.1). The first two species are annotated and have assembled chromosomes, whereas the latter two species do not. The latter two species were not included in our taxon sampling either. Therefore, here we focused our analyses on *M. asiaticus* and *X. texanus*. For each species, we searched the gene copy sequences of each of the five nuclear genes one by one against the corresponding annotated reference genome using the National Center for Biotechnology Information (NCBI) BLASTn tool. The chromosome where each gene copy is located, the range, and the percent identity (percentage of the nucleotides that are the same between the two sequences) between our sequences and the corresponding gene copy sequences from the genomes were recorded. We also searched the genome of zebrafish (*Danio rerio*) to find the location of each of the five nuclear genes we used.

### 2.9. Selection Tests

Codon-based selection tests were performed, and alignment-wide dN/dS values were calculated for nuclear gene copies of Catostomidae and its lineages using the program HyPhy v.2.5.62 [52]. Such analyses were not performed on some lineages or gene copies because they either do not contain enough sequences (i.e., Myxocyprininae and Cycleptinae) or contain indels in the sequences that disrupt their original reading frames (see later in Results). The model “MG94CUSTOMCF3X4” was used for all analyses, following suggestions in the tutorial of HyPhy. Branch lengths were estimated independently by maximum likelihood. Other model parameters were shared by all branches. The GTR mutation model in the MG94 model was chosen. For each dataset, the subtree for each lineage was extracted from the best ML tree we built earlier for the entire dataset.

### 2.10. Check Read Length Needed for Correct Assembling

The five nuclear gene datasets we assembled in this study, especially the data collected through the DNA cloning method, gave us a great opportunity to evaluate the read length requirement for correct assembling of gene copies and alleles. This information is important for future studies on Catostomidae and other taxonomic groups when people plan to collect DNA sequence data from more nuclear genes. For each species that we obtained both copies of a nuclear gene, we calculated the distance (in base pairs) between neighboring variable sites of the two copy sequences. For a RAG1 gene copy that has two allele sequences, one sequence was randomly picked to represent that gene copy. The calculations were performed in R v.4.3.1 [53] and the results for each nuclear gene were compiled and plotted using the “stripchart” function of the package *graphics* v4.3.1. The scripts we used can be found on GitHub (https://github.com/LeiYang-Fish). For RAG1, we obtained both allele sequences of a gene copy for some species using the DNA cloning method. The distance between neighboring variable sites of the two allele sequences were also calculated and plotted for each species.

## 3. Results

### 3.1. Mitochondrial Phylogeny

**Figure 1:**
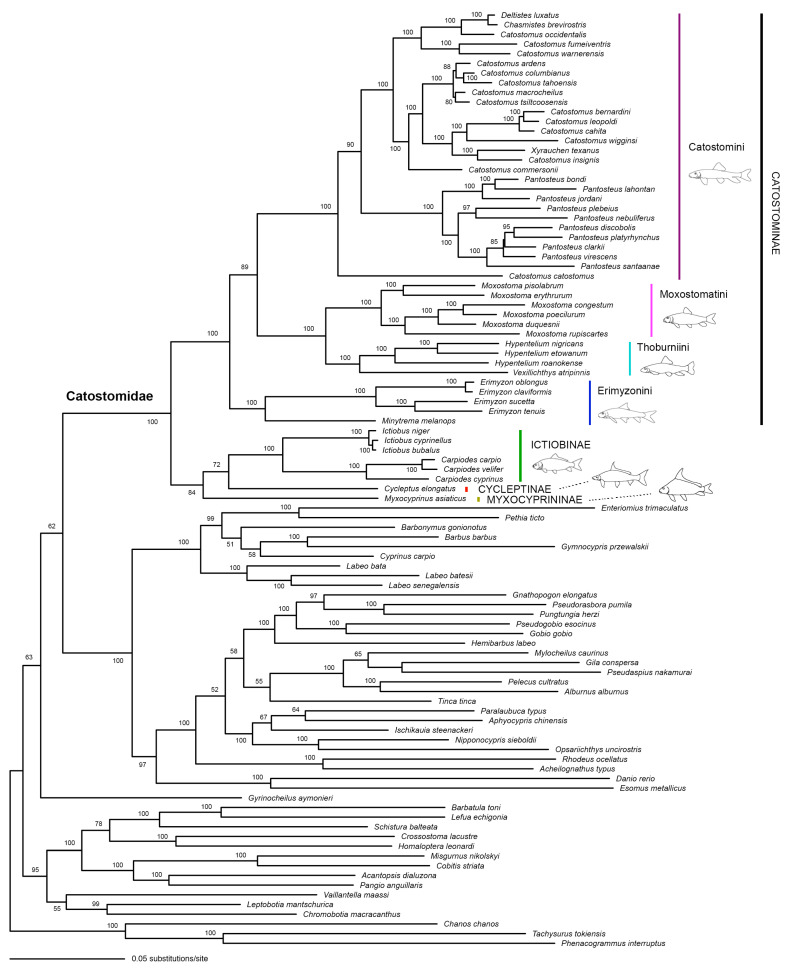
The best Maximum Likelihood tree (−ln*L* = 354,866.584470) built based on the Mitochondrial dataset. Numbers beside nodes are bootstrap support values (BP). Only those values ≥ 50% are shown.

In the tree built based on the Mitochondrial dataset (Figure 1), all subfamilies and tribes were resolved as monophyletic with BP = 100% (Myxocyprininae is monotypic, and we only analyzed one of the two species of Cycleptinae). Non-Catostominae subfamilies (Myxocyprininae, Cycleptinae, and Ictiobinae) formed a monophyletic group (BP = 84%) and the latter two are sister to each other (BP = 72%). Within Catostominae, the tribes Moxostomatini and Thoburnini formed a sister group (BP = 100%). Moxostomatini/Thoburnini is sister to Catostomini with BP = 80%.

### 3.2. Nuclear Gene Copies and Phylogenies

**Figure 2:**
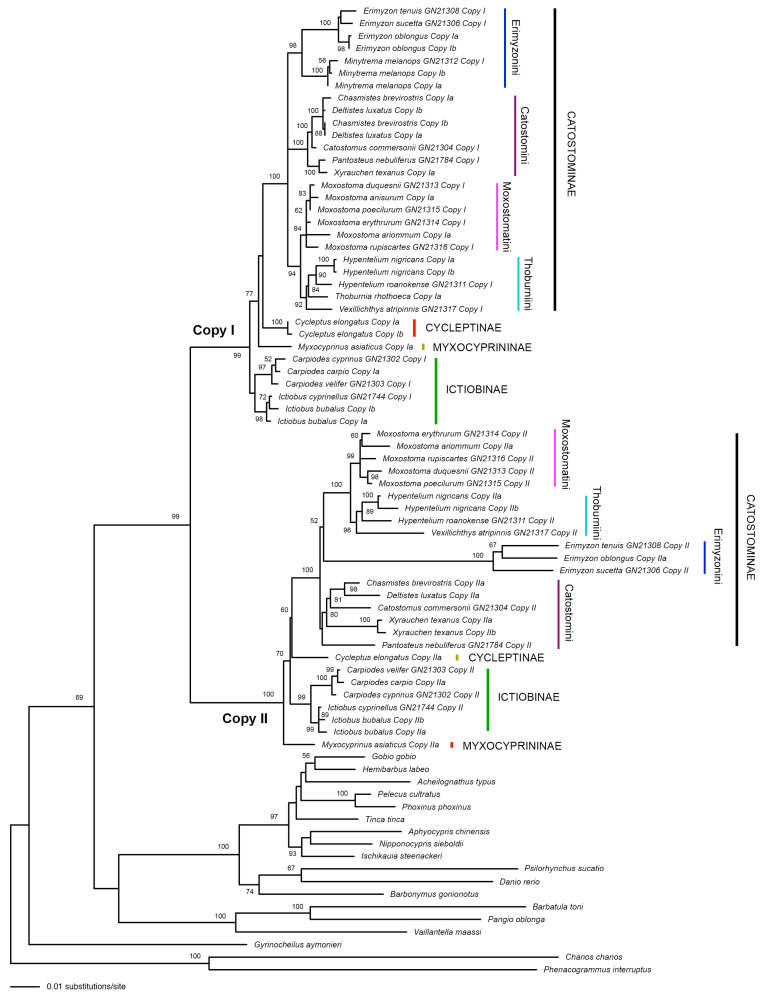
The best Maximum Likelihood tree (−ln*L* = 14,702.798774) from partitioned analysis on the RAG1 dataset. Numbers beside nodes are bootstrap support values (BP). Only those values ≥ 50% are shown. “Copy I” and “Copy II” are the two copies of the RAG1 fragment. For samples whose names contain “GN” numbers, we used “Method 2” (Next-Generation Sequencing followed by data phasing) to assemble the gene copies. Alleles of a gene copy were not separated. For samples whose names contain no “GN” numbers, we used “Method 1” (DNA cloning followed by data phasing) to assemble the gene copies and alleles (denoted by “a” and “b”). Note that we have used both methods for the species *Minytrema melanops*.

**Figure 3:**
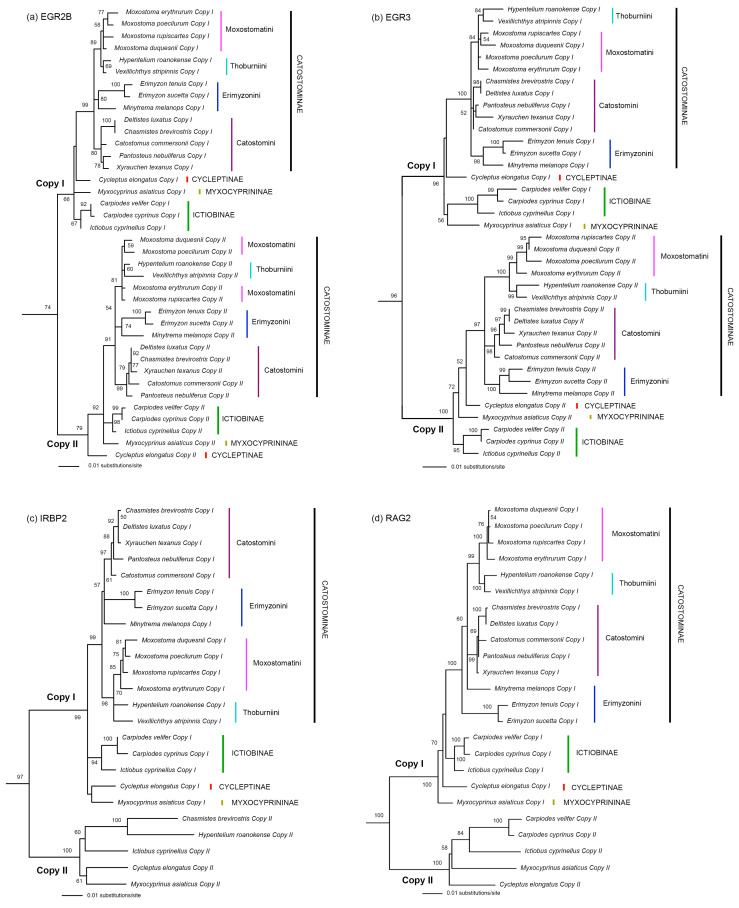
The best Maximum Likelihood trees from partitioned analysis on the four other nuclear gene datasets. Outgroups are excluded Numbers beside nodes are bootstrap support values (BP). Only those values ≥ 50% are shown. “Copy I” and “Copy II” are the two copies of each gene. “Copy I” and “Copy II” were named arbitrarily with no indication on their locations on subgenomes. The gene copy with more indels was usually named “Copy II”. (**a**) EGR2B tree (−ln*L* = 7808.300904); (**b**) EGR3 tree (−ln*L* = 6369.469377); (**c**) IRBP2 tree (−ln*L* = 7346.823013); (**d**) RAG2 tree (−ln*L* = 8573.609660).

We have successfully assembled the two gene copies (Copy I and Copy II) in Catostomidae for each of the five nuclear genes (RAG1, EGR2B, EGR3, IRBP2, and RAG2) analyzed in this study (Figure 2 and Figure 3). “Copy I” and “Copy II” were named arbitrarily with no indication on their locations on subgenomes. The gene copy with more indels was usually named “Copy II”. For EGR2B and EGR3, we have both gene copy sequences for all the catostomid species analyzed. For RAG1, IRBP2, and RAG2, the Copy I sequences have been assembled for all the analyzed catostomids, but the Copy II sequences have only been successfully assembled for some of the species and many of them contain indels and unexpected stop codons (Table 2). In each tree built based on an individual nuclear gene dataset, all Copy I sequences formed a clade that is sister to the clade formed by all Copy II sequences (Figure 2 and Figure 3).

In the RAG1 tree (Figure 2), for both Copy I and Copy II, the monophyly of nearly all major tribes and subfamilies and the sister relationships between the tribes Moxostomatini and Thoburnini are well-supported (BP ≥ 84%), except that the Copy I part of Ictiobinae and the Copy II part of Catostomini are poorly supported as monophyletic (BP < 50%). The non-Catostominae subfamilies did not form a monophyletic group in the tree for both gene copies. The relationships among Moxostomatini/Thoburnini, Erimyzonini, and Catostomini were not resolved by Copy I. In the gene tree portion of Copy II, Erimyzonini is sister to Moxostomatini/Thoburnini with a bootstrap support of only 52%.

In the EGR2B tree, EGR3 tree, IRPB2 tree, and RAG2 tree, the subfamily Catostominae is usually highly supported as monophyletic for both gene copies (Figure 3). We ignored the Copy II part of both the IRBP2 tree and the RAG2 tree, because each of them contains only five species (Figure 3c,d). The three non-Catostominae subfamilies did not form a monophyletic clade in any of the nuclear trees like they did in the mitochondrial tree. The monophyly of the subfamily Ictiobinae, the tribe Catostomini, and the group formed by Moxostomatini and Thoburniini are constantly supported as monophyletic across gene trees. The monophyly of Moxostomatini is supported by EGR2B Copy I, EGR3 Copy II, IRBP2 Copy I, and RAG2 Copy I. The tribe Erimyzonnini is monophyletic in the EGR2B tree and the EGR3 tree, but not in the Copy I part of the IRBP2 tree and the RAG2 tree. The relationships among Moxostomatini/Thoburnini, Erimyzonini, and Catostomini were either not resolved or only weakly supported in any of the four nuclear gene trees.

### 3.3. Phylogenies Built Based on the Concatenated Datasets

**Figure 4:**
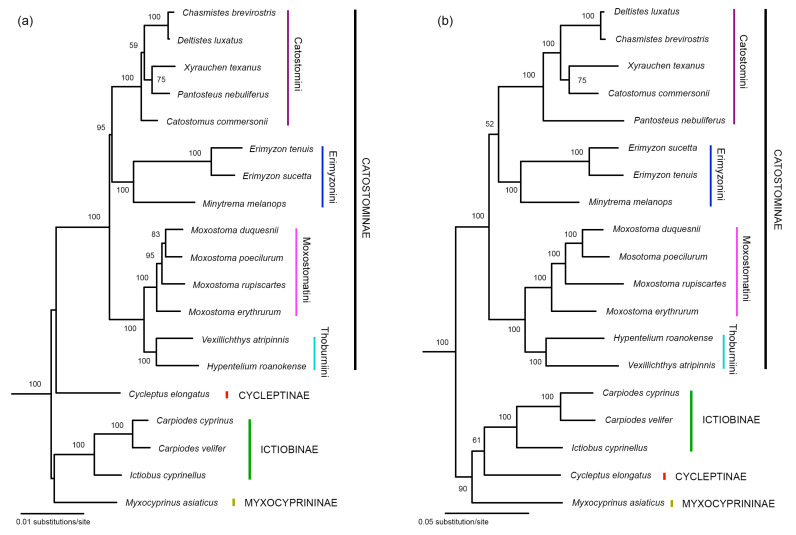
The best Maximum Likelihood tree built based on: (**a**) the 7-nuclear dataset (−ln*L* = 162,864.583786) and (**b**) the All-gene dataset (−ln*L* = 206,768.009482). Outgroups are excluded. Numbers beside nodes are bootstrap support values (BP). Only those values ≥ 50% are shown.

The monophyly of the family Catostomidae and all major subfamilies and tribes (except for Myxocyprininae and Cycleptinae that each contains only one species in the trees) are supported by both the 7-nuclear gene tree and the All-gene tree (Figure 4). The non-Catostominae subfamilies formed a monophyletic clade in the All-gene tree but not in the 7-nuclear gene tree. Catostomini is siter to Erimyzonini in both trees, although this relationship received higher support in the 7-nuclear gene tree (BP = 95%) than in the All-gene tree (BP = 52%).

### 3.4. Nuclear Genes’ Locations on Genomes

The chromosome where each gene copy is located, the range of the targets, and the percent identity between our sequences and the corresponding GenBank sequences can be found in Table 3. The gene copies are located on six different chromosomes (24, 32, 33, 36, 46, and 48) of *M. asiaticus* and six different chromosomes (27, 33, 37, 40, 46, and 49) of *X. texanus*. The values of percent identity range from 98.13% to 100.00%. Although we did not obtain the Copy II sequences of the IRBP2 gene and the RAG2 gene in our *X. texanus* sample, we were able to find their chromosome locations (33 and 49, respectively) based on the blasting results of their corresponding Copy I sequences (Table 3). The location of each of the five nuclear genes on the zebrafish genome was also shown (Table 3).

### 3.5. Selection Test Results

The calculated dN/dS values are very small (<0.2) for nearly all gene copies of all lineages tested (Table 4). It is 0.269 for the Copy I alignment of the RAG1 gene in the tribe Thoburniini and 0.491 for the Copy I alignment of the RAG2 gene in the tribe Moxostomatini.

### 3.6. Read Length Needed for Correct Assembling

**Figure 5:**
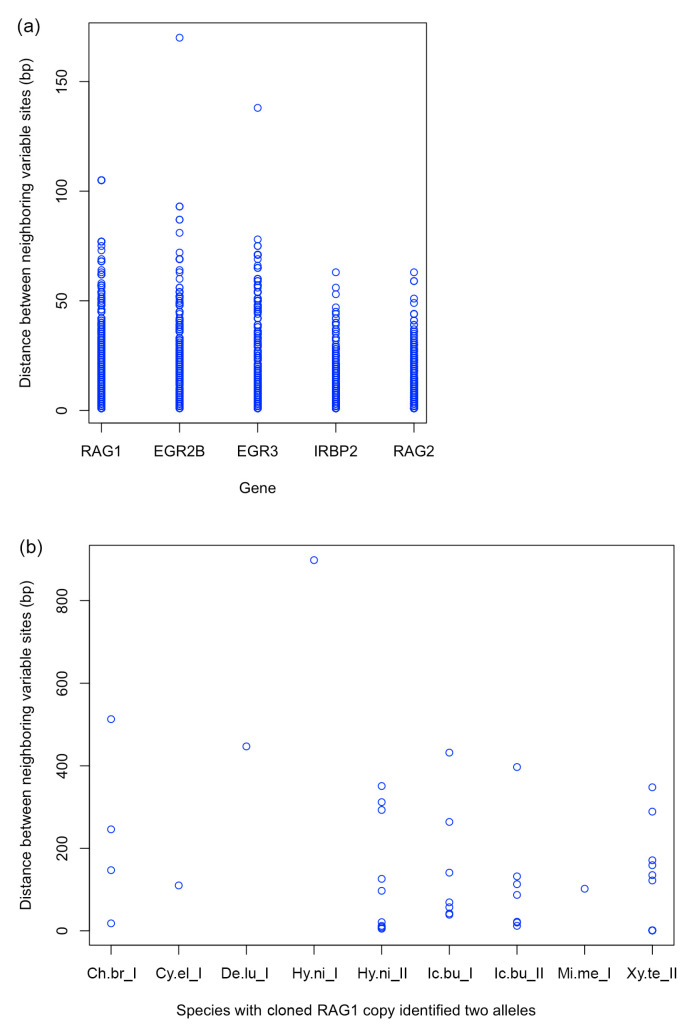
Plots showing distances (in base pairs) between neighboring variable sites of nuclear genes. (**a**) Plot by gene for species with sequences for both gene copies. For a RAG1 gene copy that has two allele sequences, one sequence was randomly picked to represent that gene copy. (**b**) Plot for RAG1 copy that we have obtained sequences for both alleles through DNA cloning.

Our results show that, for the five nuclear genes, most distances between neighboring variable sites of gene copy pairs are shorter than 100 bp (Figure 5a). In three cases, that distance is longer than 100 bp and in one case longer than 150 bp. The distances between neighboring variable sites of RAG1 allele pairs are longer than 200 bp in many cases, with the longest one being over 800 bp in length (Figure 5b).

## 4. Discussion

### 4.1. Nuclear Gene Copies and Polyploidy Evolution in Catostomidae

Most previous studies that used nuclear genes to study the phylogenetic relationships of the Catostomidae did not separate the different gene copies from these tetraploid fishes (e.g., [22,23]). Bart et al. (2010) studied the evolution of the growth hormone gene in some species of catostomid fishes [16]. They did separate two copies of that gene. However, one of the gene copies did not form a monophyletic group in their tree, indicating that the nuclear gene they used is likely not single-copy but rather from a gene family. In the present study, we successfully separated both copies of five nuclear genes for catostomid species. For each gene, sequences from the two copies formed two reciprocally monophyletic clades in the tree (Figure 2 and Figure 3).

In this study, we used two methods to separate nuclear gene copies: DNA cloning followed by data phasing and Next-Generation Sequencing followed by data phasing. The second method is new and has been proved especially effective. The RAG1 sequence we obtained using the second method for *Minytrema melanops* matched with the sequence obtained using the first method for a different individual of the same species (see Figure 2). The sequences obtained using the second method for five nuclear genes of *Myxocyprinus asiaticus* and *Xyrauchen texanus* also matched well with corresponding sequences in the reference genomes of the two species (Table 3). It should be noted that both methods we used here require all gene copies be successfully amplified by PCR first. Sometimes, mutations occur at the primer binding region of a gene copy. If the PCR amplicons contain only one gene copy, only the sequence of that gene copy will be obtained by either method. For example, we noticed that the primer set R1 2533F & R1 4090R only amplified the Copy I of the RAG1 gene in the tribes Moxostomatini, Thoburnini, and some species of Catostomini. We tried another two reverse primers (R1 4061R and R1 4078 R) to pair with R1 2533F for PCR amplifications and finally obtained the Copy II sequences for more species. PCR amplification efficiency may vary across different gene copies. This should be less an issue for the NGS-based method than the cloning-based method, because the former method can easily get hundreds of times of reads than the latter method. The Copy II of RAG1, IRBP2, and RAG2 in some species may have been lost completely and thus cannot be amplified by PCR. In Table 2, we noticed that some indels are unique to individual species, whereas others are shared among several species (e.g., the three species of *Erimyzon* in the RAG1 dataset). That means some mutations happened after the speciation of a species, whereas other mutations happened in the common ancestor of several species.

The distribution patterns of indels and unexpected stop codons and the selection tests results together showed that one gene copy of RAG1, IRBP2, and RAG2 were apparently under more relaxed purifying selection and evolving faster than the other copy of the corresponding gene (Table 2 and Table 4). In other words, one gene copy has dominance over the other gene copy for these genes. It will be interesting to investigate whether this pattern of asymmetry persists at the subgenome level (subgenome dominance). If so, it will mirror the situation seen in the allotetraploid African clawed frog [54] and many polyploid plants [55].

Uyeno and Smith (1972) hypothesized that species in the family Catostomidae diversified from a single tetraploid ancestor [13]. Results from our analyses of five nuclear gene datasets (Figure 2 and Figure 3) are consistent with this suggestion and indicate that the tetraploidization of this group of fishes occurred during the formation of their most recent common ancestor. However, we are not sure if that tetraploidization event happened only once or recurrently (e.g., [56,57]). Uyeno and Smith (1972) also hypothesized that Catostomidae have diversified from their most recent common ancestor since at least 50 Ma in the early Eocene [13]. They hypothesized that catostomid fishes evolved from a diploid cyprinid-like ancestor and hinted that the tetraploidization of the common ancestor of catostomids may be preceded by hybridization. Ferris (1984) also believed that allopolyploidization is the origin mode of the Catostomidae [58]. The recent study on the whole genome sequence of *Myxocyprinus asiaticus* clearly supported the allopolyploid origin of this species [24,25]. In our nuclear gene trees (Figure 2 and Figure 3), each gene copy formed a clade, and the two clades are sister to each other. The apparent divergence between the two copies of each gene seems consistent with the allotetraploid origin of this family. However, the maternal progenitor and paternal progenitor of the tetraploid ancestor of Catostomidae cannot be traced like what have been done in some cyprinid fishes (e.g., [14]) and they likely went extinct. They may be cyprinid-like fishes but are unlikely members of the family Cyprinidae, because no cyprinid species are located within, or constitute the sister group of any of the two clades of the Catostomidae in the tree. Data from more and longer nuclear genes are needed to further explore the polyploidy evolution of the family Catostomidae.

### 4.2. Phylogenetic Relationships Among Major Lineages

In this study, we not only built a mitochondrial tree to reflect the maternal relationships among major lineages of catostomids, but also tried to show the relationships among these lineages as revealed by both the maternal and paternal copies of five nuclear genes. Comparing with previous studies (e.g., [19,21]) our mitochondrial analyses were performed based on more whole mitogenome sequences and provided a better resolution for the maternal relationships among subfamilies/tribes of the family Catostomidae (Figure 1). Moreover, unlike previous studies, we have separated the two gene copies for five single-copy nuclear genes and built gene trees to show relationships among major lineages of Catostomidae (Figure 2 and Figure 3).

As we mentioned earlier, there are currently two major disagreements among studies regarding the phylogenetic relationships among subfamilies/tribes of Catostomidae. The first disagreement lies in whether Myxocyprininae, Cycleptinae, and Ictiobinae constitute a monophyletic group and their relationships with Catostominae. The second disagreement is whether Catostomini is sister to Moxostomatini/Thoburniini or Erimyzonini or a clade formed by them. Our mitochondrial tree (Figure 1) and All-gene tree (Figure 4b) both support that Ictiobinae, Cycleptinae, and Myxocyprininae formed a monophyletic group (BP = 84% and 90%, respectively) and the first two subfamilies are sister to each other (BP = 72% and 61%, respectively). Our nuclear trees (including the 7-nuclear tree) generally did not resolve the relationships among Ictiobinae, Cycleptinae, Myxocyprininae, and Catostominae very well (Figure 2, Figure 3 and Figure 4). However, the possibility that the three non-Catostominae subfamilies formed a monophyletic group cannot be ruled out due to the low bootstrap support on the nodes. The fact that adding nuclear data to the mitochondrial data improved the node support (from 84% to 90%) of a monophyletic non-Catostominae clade renders us to believe that it is very likely monophyletic. There is noise in the nuclear data, which may be the consequence of incomplete lineage sorting. To further test this hypothesis, more data from the nuclear genome will be needed.

The mitochondrial tree support the sister relationship between Catostomini and Moxostomatini/Thoburniini (BP = 89%; Figure 1). The five individual nuclear gene trees generally failed to resolve the relationships among the three tribes of Catostominae (Figure 2 and Figure 3). Surprisingly, the 7-nuclear tree support a sister relationship between Catostomini and Erimyzonini (BP = 95%; Figure 4a). The All-gene tree also support this relationship but with a much lower bootstrap value (52%), which clearly reflect the conflict between signals from the mitochondrial genes and the nuclear genes (Figure 4b). We also suggest using more data from the nuclear genome to help find out the source of conflict between gene trees.

It is not an easy task to resolve phylogenetic relationships among polyploids. In many cases, phylogenetic relationships among polyploids cannot be fully shown by a single bifurcated tree due to complicated reticulate evolution (e.g., [14]). In Catostomidae, however, we do not have to deal with reticulate evolution, because the polyploidization event happened in the common ancestor of all members of this family. For a diploid group, the incongruence among gene trees may be caused by various biological factors (e.g., incomplete lineage sorting, horizontal gene transfer) and analytical factors (e.g., model misspecification; [59,60]). For a tetraploid group like Catostomidae, besides all that, we also need to deal with the fact that there are two sets of nuclear genes in the genome and the evolution of one copy of a gene may be affected by the existence of the other copy of the same gene. For example, the Copy II sequences of RAG1 were under relaxed purifying selection, which may have affected the topology and branch lengths of the tree (see the long branch leads to *Erimyzon* in Figure 2). That is why we ignored the Copy II of RAG1, IRBP2, and RAG2 when it comes to discussion on phylogenetic relationships.

Because most previous studies on the phylogeny of Catostomidae either did not use nuclear genes or used nuclear genes but did not separate the gene copies, we have thought that paralogy issue may have contributed significantly to the disagreements among studies. In the current study, we separated the gene copies for five single-copy nuclear genes, which should largely eliminate the paralogy issue caused by either whole genome duplication or small-scale gene duplication [17]. However, the disagreements among nuclear gene trees and between nuclear gene trees and mitochondrial gene trees persist. It seems evident now that other factors (e.g., incomplete lineage sorting) may have caused the disagreements among gene trees and among studies.

### 4.3. Nuclear Genes’ Locations on Genomes and Implications

Our results showed that the Copy I of RAG1 and RAG2 is located on the same chromosome (#46), and the Copy II of these two genes is also located on the same chromosome (#48 for *M. asiaticus*; #49 for *X. texanus*; see Table 3). The Copy I of EGR2B and IRBP2 is on the same chromosome (#36 for *M. asiaticus*; #32 for *X. texanus*), and the Copy II of these two genes is also on the same chromosome (#40 for *M. asiaticus*; #33 for *X. texanus*). The Copy I and Copy II of EGR3 is not located on the same chromosomes (#33 & #24 for *M. asiaticus*; #37 & #27 for *X. texanus*) as RAG1/RAG2 and EGR2B/IRBP2. In zebrafish (*Danio rerio*), which is a diploid and each of these genes only has a single gene copy, RAG1 is also on the same chromosome (#25) as RAG2, EGR2B is on the same chromosome (#12) as IRBP2, but EGR3 is on the chromosome 8 (Table 3). Chromosomes are usually numbered according to their sizes. The fact that same gene copies are found on different chromosomes in *M. asiaticus* and *X. texanus* can at least partly reflect variations in genomic dynamics across the two species. It is possible that genomic recombination has played a role, but a detailed genomic level analysis is needed, which is not the focus of this study. According to Table 3, the RAG1 fragment and the RAG2 fragment we used in this study are very close to each other (~2300 bp) on the chromosomes, but the EGR2B fragment and the IRBP2 fragment are far from each other (>5 million bp). This can help explain why there are many indels, unexpected stop codons, and possible gene loss in the Copy II of both RAG1 and RAG2. They are in the same gene complex and behaved like a single gene. On the contrary, EGR2B and IRBP2 behaved like two completely independent genes. There are many indels, unexpected stop codons, and possible gene loss in the Copy II of IRBP2, but none of them were found in the Copy II of EGR2B.

### 4.4. Implications for Future Studies

Based on our results, more genetic information from the nuclear genome is needed to better resolve the relationships among major subfamilies and tribes of the Catostomidae. We may need to obtain DNA sequences from hundreds of nuclear gene fragments using gene capture or other techniques (e.g., whole genome sequencing). We hope what we presented in this study can contribute to the resolving of the phylogenetic relationships among suckers eventually. Sperstad (2018), which is a master thesis, studied the phylogenetic relationships and evolution of the Catostomidae using DNA sequences from a few hundred nuclear loci generated through anchored hybrid enrichment [61,62]. In Sperstad (2018), the author claimed that “This phasing process resulted in each of the 43 catostomids being represented by four alleles at each locus…” [61]. However, because the Vertebrate v.1 kit used by Sperstad (2018) was designed to target conserved gene regions and the study used the Illumina HiSeq 2000 platform for sequencing and the read length is only 100 bp, there is a high chance that some allele or paralog sequences of some nuclear loci generated by the study are actually chimeric sequences, which can mislead the results of phylogenetic and evolutionary analyses performed. We showed in the present study (Figure 5) that assembling NGS data with a read length of 100 bp will very likely create chimeric sequences for some gene copies and most alleles. For future studies on the phylogenetics and evolution of Catostomidae, we suggest using either long-read sequencing (e.g., PacBio or Oxford Nanopore; [6,26]) or short-read sequencing with relatively longer read length (e.g., 300 bp) to separate gene copies. Alleles of gene copies should only be separated using long-read sequencing.

## 5. Conclusions

Studying the phylogenetic relationships of polyploids has been challenging due to difficulties in separating paralogous nuclear gene copies. In this study, we investigated the phylogenetic relationships of the tetraploid fish family Catostomidae, using both mitochondrial and nuclear genes. We employed two different methods to separate gene copies of five nuclear genes: DNA cloning followed by data phasing and Next-Generation Sequencing followed by data phasing. The latter method, newly developed in this study, proved especially effective. As part of this method, we introduced the R package *copyseparator* for the first time. Our analyses support Uyeno and Smith’s (1972) hypothesis that species in the family Catostomidae diversified from a single tetraploid ancestor [13]. The data also support a sister relationship between Catostominae and a monophyletic group formed by Myxocyprininae, Cycleptinae, and Ictiobinae. Within Catostominae, however, there is disagreement between mitochondrial and nuclear data regarding the relationships among Erimyzonini, Catostomini, and Moxostomatini/Thoburnini. For future studies, we suggest sequencing additional nuclear genes using either long-read sequencing or short-read sequencing with relatively longer read lengths to minimize the creation of chimeric sequences.

## Figures and Tables

**Table 1:** Taxon sampling and characteristics of different datasets used in this study.

	Mito	RAG1	EGR2B	EGR3	IRBP2	RAG2	7-Nuclear	All-Gene
Catostomidae	51	26	19	19	19	19	19	19
Outgroup	46	18	26	17	17	10	15	15
Total species	97	44	45	36	36	29	34	34
Total sequences	97	78	64	55	41	34	34	34
Nucleotides (bp)	14,888	1497	846	953	864	1315	7237	22,125
Variable characters (bp)	8099	830	362	356	499	676	2897	10,084
Parsimony-informative characters (bp)	6929	616	318	271	331	418	1907	7835
A%	27.6	26	22.5	24.4	27.1	24.6	24.6	26.8
C%	26.2	24.2	37.1	33.3	24.7	25.9	30.1	27.5
G%	18.5	26.3	22	23.1	23.8	25.2	24.1	20.2
T%	27.7	23.5	18.4	19.2	24.4	24.3	21.2	25.5

**Table 2:** Indels and unexpected stop codons found in the different copies of nuclear genes. Their locations in each gene alignment are shown.

Gene	Species	Copy	Indel	Unexpected Stop Codon
RAG1	*C. commersonii*	II	628–634	574
	*C. brevirostris*	IIa		1015; 1093
	*D. luxatus*	IIa	738–740; 1345–1350	
	*E. oblongus*	IIa	82–94; 127; 265; 366–396; 435–440; 504–507; 702–705; 849–854; 910–969; 1106–1108; 1147	322; 823; 1015; 1084
	*E. sucetta*	II	11; 265; 366–396; 435–440; 504–507; 702–705; 795–797; 966–970; 1053–1058; 1147; 1371–1372	73; 322; 823; 856; 952; 1015
	*E. tenuis*	II	82–94; 265; 366–396; 435–440; 504–507; 702–705; 966–970; 1145–1147; 1371–1372	322; 823; 856; 952; 1015
	*H. roanokense*	II	706–711	
	*P. nebuliferus*	II	965	10
	*V. atripinnis*	II		1195
	*X. texanus*	IIa	204–214; 903–907; 987; 1054–1070; 1345	106
	*X. texanus*	IIb	903–907; 987; 1054–1070; 1345	106
EGR3	*C. brevirostris*	I	219–221	
	*C. elongatus*	II	192–197	
	*P. nebuliferus*	I	192–197	
IRBP2	*C. brevirostris*	II	45–57; 299–300; 432–440; 584–586; 642; 856	510; 594
	*C. elongatus*	II	495	231
	*H. nigricans*	II	45–57; 299–300; 856	
	*I. cyprinellus*	II	344	
	*M. asiaticus*	II		279
RAG2	*C. cyprinus*	II	1082; 1292 (insertion)	158; 176; 230; 482; 728; 890; 926
	*C. velifer*	II	1082; 1292 (insertion)	158; 176; 230; 482; 728; 890; 926
	*C. elongatus*	II	394; 1082; 1295	176; 230; 398; 728; 890; 1046
	*I. cyprinellus*	II	921–947; 1082; 1292 (insertion)	158; 176; 230; 398; 728; 890; 950; 1265
	*M. asiaticus*	II	527; 995–1000; 1082	177; 230; 404; 482; 575; 728; 890

Note: No indel or unexpected stop codon was found in any EGR2B sequence.

**Table 3:** Results of blasting against the nuclear reference genome sequences of *Myxocyprinus asiaticus* (GenBank: GCA_019703515.2) and *Xyrauchen texanus* (GenBank: GCA_025860055.1), respectively. The gene copy sequences obtained by us for each nuclear gene of the corresponding species were used as queries. “Copy I” and “Copy II” were named arbitrarily with no indication on their locations on subgenomes. The five genes’ chromosome locations on the genome of the diploid zebrafish (*Danio rerio*) are also shown.

Gene	Zebrafish Chromosome	Species	Copy	Chromosome	Range	Percent Identity
RAG1	25	*M. asiaticus*	Copy I	46	7,634,270–7,635,766	99.93%
			Copy II	48	23,625,477–23,626,973	100.00%
		*X. texanus*	Copy I	46	21,763,507–21,765,003	98.13%
			Copy II	49	5,888,675–5,890,147	98.91%
RAG2	25	*M. asiaticus*	Copy I	46	7,638,033–7,639,346	100.00%
			Copy II	48	23,621,928–23,623,233	100.00%
		*X. texanus*	Copy I	46	21,759,909–21,761,222	100.00%
			Copy II	49 *^#^	5,891,844–5,892,189	NA
EGR2B	12	*M. asiaticus*	Copy I	36	35,497,550–35,498,366	99.75%
			Copy II	32	8,186,002–8,186,818	100.00%
		*X. texanus*	Copy I	40	30,913,549–30,914,365	100.00%
			Copy II	33	7,218,725–7,219,541	99.75%
IRBP2	12	*M. asiaticus*	Copy I	36	29,737,521–29,738,384	99.77%
			Copy II	32	14,358,706–14,359,569	99.65%
		*X. texanus*	Copy I	40	25,306,591–25,307,454	100.00%
			Copy II	33 *	12,975,085–12,975,913	NA
EGR3	8	*M. asiaticus*	Copy I	33	10,026,197–10,027,149	100.00%
			Copy II	24	38,144,752–38,145,704	100.00%
		*X. texanus*	Copy I	37	9,175,709–9,176,661	99.90%
			Copy II	27	8,566,123–8,567,075	99.79%

* No Copy II sequence was obtained by us. The Copy I sequence was used as query. ^#^ Only 346 bp (26.33%) of the query sequence found target on the Chromosome 49. NA: not available.

**Table 4:** Selection test results for different gene copies of different lineage.

Lineage	RAG1_I	IRBP2_I	RAG2_I	EGR2B_I	EGR2B_II	EGR3_I	EGR3_II
Catostomidae	0.115	0.167	0.165	0.031	0.022	0.079	0.034
Catostominae	0.132	0.188	0.179	0.029	0.024	0.058	0.042
Catostomini	0.091	0.072	0.164	0.000	0.062	0.143	0.000
Erimyzonini	0.043	-	-	0.022	0.029	0.057	0.034
Moxostomatini	0.491	0.065	0.196	0.027	-	-	0.000
Thoburniini	0.16	-	0.269	0.126	0.000	0.000	0.056
Ictiobinae	0.024	0.089	0.064	0.061	0.000	0.074	0.000

## Data Availability

All sequence data used in this study can be found in GenBank (accession numbers are provided in Tables S1 and S2).

## Supplementary Materials

The following supporting information can be downloaded at: https://www.mdpi.com/article/10.3390/biology13121072/s1, Figure S1: Key steps of the pipeline used by *copyseparator* to assemble long gene copies from short read data; Table S1: Mitochondrial dataset sample information; Table S2: Nuclear dataset sample information.
