# Supplementary material for: Phylogeny and Polyploidy Evolution of the Suckers (Teleostei: Catostomidae)
Source: Biology (Basel). 2024 Dec 20;13(12):1072. doi: 10.3390/biology13121072 (PMC11673241; doi:10.3390/biology13121072)
Supplement: Supplementary file 1 [file biology-13-01072-s001.zip › Figure S1 Key steps of the pipeline used by copyseparator to assemble long gene copies from short read data.pdf]

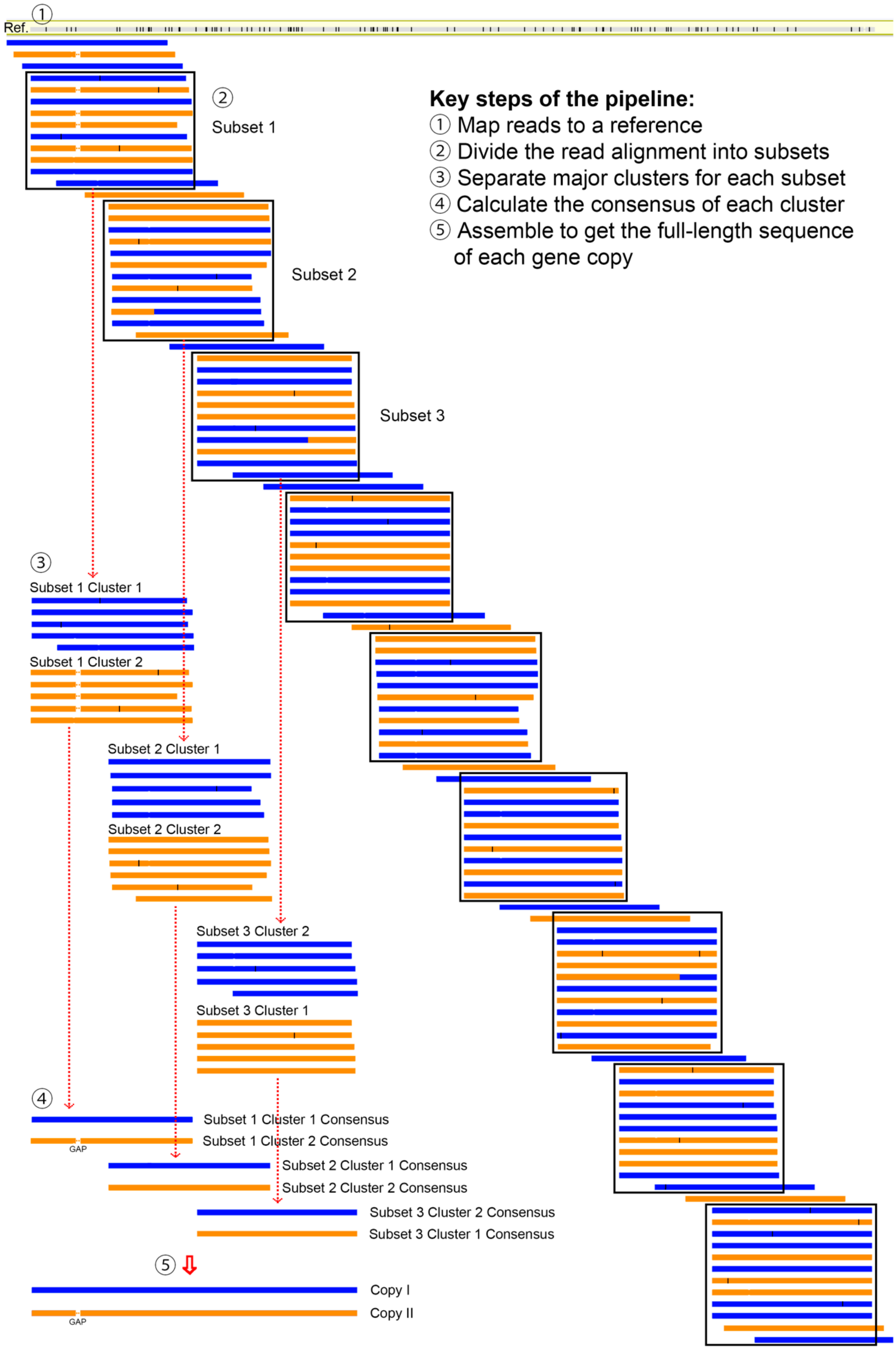

Ref.
Key steps of the pipeline:
① Map reads to a reference
② Divide the read alignment into subsets
③ Separate major clusters for each subset
④ Calculate the consensus of each cluster
⑤ Assemble to get the full-length sequence of each gene copy
Subset 1
Subset 2
Subset 3
Subset 1 Cluster 1
Subset 1 Cluster 2
Subset 2 Cluster 1
Subset 2 Cluster 2
Subset 3 Cluster 2
Subset 3 Cluster 1
Subset 1 Cluster 1 Consensus
Subset 1 Cluster 2 Consensus
GAP
Subset 2 Cluster 1 Consensus
Subset 2 Cluster 2 Consensus
Subset 3 Cluster 2 Consensus
Subset 3 Cluster 1 Consensus
Copy I
Copy II
GAP

Fig. S1 Key steps of the pipeline used by *copyseparator* to assemble long gene copies from short read data. First, short reads are mapped to a reference (Step 1). Reads that are not directly mapped to the reference and the reference itself need to be removed after mapping. Second, the large read alignment is divided into multiple (9 in this figure) subsets that have significant overlaps between neighboring subsets (Step 2). After that, sequences that are empty or too short are removed. Each bar, either blue or orange, showed in this figure may represent dozens or even hundreds of reads in reality. From Step 3 to Step 5, the first three subsets are used to illustrate how to get the full length of each gene copy. We first separate major clusters for each dataset (Step 3). We then calculate to get the consensus sequence for each cluster of each subset (Step 4). At last, we assemble the sequences from the last step to get the full-length sequence of each gene copy (Step 5). As can be seen from the figure, the number of subsets can be a large number and the full length of each gene copy can thus be very long. Short indels in gene copies seem have no impact on the assembling process.
